# Sodium/potassium ratio change was associated with blood pressure change: possibility of population approach for sodium/potassium ratio reduction in health checkup

**DOI:** 10.1038/s41440-020-00536-7

**Published:** 2020-08-17

**Authors:** Mana Kogure, Naoki Nakaya, Takumi Hirata, Naho Tsuchiya, Tomohiro Nakamura, Akira Narita, Yoko Suto, Yoko Honma, Hidemi Sasaki, Ken Miyagawa, Yusuke Ushida, Hiroyuki Ueda, Atsushi Hozawa

**Affiliations:** 1 Division of Personalized Prevention and Epidemiology, Tohoku Medical Megabank Organization, Tohoku University, Sendai, Japan; 2 Progressive Innovation Research Project (COI Tohoku Site), Center for Promotion of Innovation Strategy, Head Office of Enterprise Partnerships, Tohoku University, Sendai, Japan; 3 Department of Health Sciences, Saitama Prefectural University, Koshigaya, Japan; 4 Department of Public Health, Hokkaido University Faculty of Medicine, Sapporo, Japan; 5 Health Promotion Division, Department of Civic Life Affairs, Tome City, Miyagi Japan

**Keywords:** Blood pressure, Community setting, Health checkup site, Population approach, Urinary Na/K ratio

## Abstract

Recently, the sodium (Na)/potassium (K) ratio was reported to be associated with blood pressure (BP). A Na/K ratio self-monitoring device using spot urine was established recently. Here, we assessed whether the urinary Na/K ratio change measured using the Na/K device was associated with BP change in a health checkup setting. We targeted 12,890 participants who attended the health checkup in Tome City, Miyagi between 2017 and 2018. Tome City introduced urinary Na/K ratio measurements during health checkups since 2017. For each year, we compared the baseline characteristics according to the urinary Na/K ratio and BP level. We assessed the relationship between change in urinary Na/K ratio and BP change using multiple regression analyses adjusted for age, sex, and change in body mass index (BMI) and alcohol intake. The average urinary Na/K ratio was significantly lower in 2018 than in 2017 (5.4 ± 3.0 to 4.9 ± 2.2, *P* < 0.01). The systolic BP of the participants in 2018 (130.9 ± 17.4 mmHg) was lower than that in 2017 (132.1 ± 17.9 mmHg). Moreover, the change in systolic BP and diastolic BP was positively associated with the change in urinary Na/K ratio. In conclusion, the association of the change in urinary Na/K ratio with hypertension and changes in systolic and diastolic BP can be explained by a change in alcohol intake, BMI, and urinary Na/K ratio. Therefore, measuring the urinary Na/K ratio in community settings is a potential population approach for counteracting hypertension.

## Introduction

Recently, it was reported that the sodium (Na)/potassium (K) ratio was associated with blood pressure (BP) in a cross-sectional analysis [1–4]. This is expected because high Na and K intake are known to be related to high and low BP, respectively [5–7]. In addition, research has been conducted on reference values and factors contributing to the Na/K ratio in the past year [8, 9].

In a community setting, it is difficult for us to assess the actual Na intake because Na intake is traditionally assessed by 24-h urine collection. Although several equations for estimating Na intake or K intake using casual spot urine were recently established, these equations are not accurate enough to provide feedback to the individuals [10–12]. Furthermore, because the equation requires measuring Na, K, and creatinine levels in a laboratory, it takes several weeks to obtain the results for individuals. Although participants are aware that salt reduction is good for lowering BP, they have no way of assessing their Na level.

Recently, a Na/K ratio self-monitoring device using spot urine was established [13]. Although it did not assess the actual Na intake level, it can provide feedback regarding the urinary Na/K balance in a few minutes. Moreover, an interventional or observational study using this monitor suggested that this might be a useful device to estimate the urinary Na/K ratio [13, 14].

Thus, although we have no way to assess the Na/K ratio in health checkup settings, we considered that assessing the urinary Na/K ratio in a health checkup site may have the potential to provide feedback to the general population regarding their Na/K ratio and might have an effect on perception change, change in action, Na/K ratio reduction, and BP reduction. In this study, we assessed whether the urinary Na/K ratio change measured with the Na/K ratio measurement device was associated with BP change independent of confounding factors such as body mass index (BMI) change and change in alcohol intake and compared the BP level and urinary Na/K ratio at the community level.

## Methods

### Participants

**Fig. 1:**
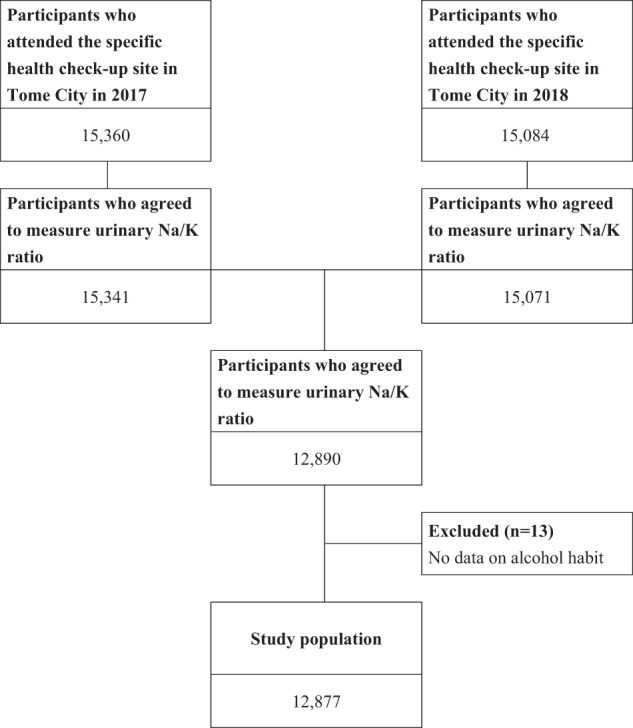
Flow chart for participant selection. In this study, we measured the urinary Na/K ratio and BP in 12,890 participants (84% of participants were from 2017) for two years. Participants with no alcohol habit data (*n* = 13) were excluded from the analyses. Finally, 12,877 participants were analyzed

In Japan, individuals with national health insurance can attend health checkups annually. The health checkups are performed according to the Health Promotion Act. In this study, we assessed the change in BP and urinary Na/K ratio in participants who underwent health checkups in Tome City, Miyagi. The city is located in the northeastern part of Miyagi Prefecture, Japan. Tome City was originally divided into nine districts and was annexed in 2005. Tome City performs health checkups in each district every year from May to September. The population of this city was approximately 78,780 in June 2019 [15]. In 2017, 15,360 participants underwent health checkups in Tome City, and a majority of these participants had National Health Insurance. The participants were asked to participate in this study, and 15,341 (99.9%) participants agreed. In 2018, 15,084 individuals were asked, and 15,071 (99.9%) participated in the study. Finally, we analyzed the urinary Na/K ratio and BP in 12,890 participants (84% of the participants were from 2017) for two years. Participants with no data for alcohol consumption (*n* = 13) were excluded. Overall, a total of 12,877 participants were included in the analysis (Fig. 1).

This study was approved by the Institutional Review Board of Tohoku Medical Megabank Organization (approval number: 2017-4-006).

### Measurements

#### Na/K ratio

Tome City asked participants to collect their urine samples after waking in the morning. The urinary Na/K ratio was measured using a urinary Na/K ratio-measuring device called HEU-001F (OMRON Healthcare Co., Ltd., Kyoto, Japan) (Supplementary Fig. 1). The monitor automatically records urinary Na/K ratio measurements. A public nurse measured the Na/K ratio from the spot urine samples of the participants using the device, recorded the value, and provided immediate feedback to the participants regarding the measured value along with explanatory material about the relationship between the urinary Na/K ratio and BP (Supplementary Fig. 2). Tome City performs health checkups in each district every year from May to September. Therefore, participants could have their urinary Na/K ratio measured at the same time in both 2017 and 2018.

#### Other measurements

Information about other measurements was obtained from the health checkup data from Tome City. Both body weight and body height were measured during the health checkup. BMI was calculated as weight (kg)/height^2^ (m^2^). BP was measured twice by a trained public health nurse. Because the health checkup data provided by Tome City only included the lower BP measurement of the two BP measurement values, we could only include this value in our study. Hypertension was defined as systolic BP (SBP) ≥ 140 mmHg or diastolic BP (DBP) ≥ 90 mmHg and/or the use of antihypertensive medication. Information on alcohol intake was obtained by a questionnaire. We calculated the alcohol intake per week as follows: we multiplied the frequency of alcohol consumption (every day = 7, sometimes = 3.5, and no habitual drinking = 0) and volume of alcohol consumed in a single occasion (0–1 drink = 0.5, 1–2 drinks = 1.5, 2–3 drinks = 2.5, and 3 drinks or more = 3) and divided the product by 7. One drink in Japan corresponds to 23 g of ethanol. Data on the use of diabetes medication and treatment for heart disease were self-reported from the health checkup data.

With regard to information on the use of diabetes medication, participants chose one option from the following responses: (a) a history of diabetes, (b) under treatment for diabetes mellitus, or (c) use of diabetes medication. Individuals who answered (c) were classified as “users of diabetes medication,” whereas those who responded with (a) or (b) or not applicable were classified as “others.” With regard to information on the treatment for heart disease, participants chose one option from the following responses: (a) have a history of heart disease or (b) undergoing treatment for heart disease. Individuals who answered (b) were classified as “undergoing treatment for heart disease,” whereas those who responded with (a) or not applicable were classified as “others.”

### Statistical analysis

To compare the difference between participants who attended health checkups both in 2017 and 2018 and those who attended health checkups only in 2017, we used the *t*-test for continuous variables and the chi-square test for categorical variables. For each year, we compared the baseline characteristics according to the urinary Na/K ratio level. Moreover, we analyzed the relationship between the urinary Na/K ratio and hypertension adjusted for age, sex, BMI, alcohol intake, use of diabetes medication, and treatment for heart disease. For longitudinal data, we assessed the relationship between change in urinary Na/K ratio and BP change using multiple regression analyses adjusted for age, sex, BMI change, and alcohol intake. In both cross-sectional and longitudinal analyses, we stratified the data according to the use of antihypertensive medication. We further added SBP or DBP in 2017 as a covariate. Furthermore, we analyzed the BP difference according to the urinary Na/K ratio quartile in 2017. To examine the effect of BP due to temperature, we acquired past temperature data from the Japan Meteorological Agency in 2017 and 2018 [16]. We considered *P* < 0.05 as statistically significant.

## Results

Table 1 shows the difference between participants who underwent health checkups both in 2017 and 2018 and those who underwent a health checkup only in 2017. Participants who did not undergo the 2018 health checkup were significantly younger, comprised more women, consumed less alcohol, had a lower urinary Na/K ratio, had lower BP, and comprised fewer users of antihypertensive medication than those who underwent the health checkup both in 2017 and 2018.

**Table 1:** Characteristics of participants both underwent 2017 and 2018 health checkup and those who visited 2017 health checkup only. We compared the characteristics of the participants who attended the health check-up site both in 2017 and 2018 and those who attended only in 2017

	Participants in 2018	Nonparticipants in 2018	*P* value
Participants in 2017			
Numbers	12,890	2,451	
Age (years) (means ± SD)	65.4 ± 13.3	61.7 ± 19.4	<0.01
Women (%)	51.0	56.7	<0.01
BMI (kg/m^2^) (means ± SD)	23.8 ± 3.6	23.6 ± 4.1^a^	0.19
Alcohol (drink/day^b^) (means ± SD)	0.5 ± 0.7^c^	0.4 ± 0.7	<0.01
Na/K ratio (means ± SD)	5.4 ± 3.0	5.3 ± 3.0	0.03
SBP (mmHg) (means ± SD)	132.1 ± 17.9	131.2 ± 20.0	0.04
DBP (mmHg) (means ± SD)	75.0 ± 11.1	73.4 ± 12.4	<0.01
User of antihypertensive medication (%)	39.4	35.5	<0.01
User of diabetes medication (%)	8.8	8.5	0.58
Treatment for heart disease (%)	6.4	7.3	0.10

*BMI* body mass index, *DBP* diastolic blood pressure, *Na*/*K* *ratio* sodium/potassium ratio, *SBP* systolic blood pressure, *SD* standard deviation

^a^*n* = 2,449

^b^Alcohol 1 drink correspond to 23 g of ethanol

^c^*n* = 12,889

Table 2 shows the characteristics of the participants who underwent health checkups both in 2017 and 2018. Supplementary Table 1 shows the characteristics of participants who underwent health checkups both in 2017 and 2018 according to urinary Na/K ratio. Both in 2017 and in 2018, a high urinary Na/K ratio was associated with older age, higher BMI, and higher SBP. Between the 2017 data and 2018 data, the average BMI and the distribution of alcohol consumption habits were unchanged. The average urinary Na/K ratio in 2018 was significantly lower than that in 2017 (5.4 ± 3.0 to 4.9 ± 2.2, *P* < 0.01). The average SBP level in 2018 was lower than that in 2017 (132.1 ± 17.9 to 130.9 ± 17.4 mmHg), but the average DBP value increased. The proportion of participants with a urinary Na/K ratio ≥ 8.0 was 14% (1,805/12,877) in 2017 and 9% (1,170/12,877) in 2018. The average temperature during the health checkup period was 20.2 °C and 20.6 °C in 2017 and 2018, respectively.

**Table 2:** Characteristics of study participants in 2017 and that in 2018, Tome Na/K Measuring Project, 2017–2018. For each year, we compared the baseline characteristics

	Participated in 2017	Participated in 2018
*n*	12,877	
Age (years)^a^	65.4 ± 13.3	66.4 ± 13.3
Sex (women)^b^	51.0	51.0
BMI (kg/m^2^)^a^	23.8 ± 3.6	23.8 ± 3.7
Nondrinker^b^	54.0	54.2
Alcohol <1 drink/day^b^	27.0	27.0
Alcohol 1–2 drink/day^b^	13.5	13.5
Alcohol 2 drink/day^b^	5.5	5.4
Na/K ratio^a^	5.4 ± 3.0	4.9 ± 2.2
SBP (mmHg)^a^	132.1 ± 17.9	130.9 ± 17.4
DBP (mmHg)^a^	75.0 ± 11.1	75.8 ± 10.9
User of antihypertensive medication^b^	39.4	41.7
User of diabetes medication^b^	8.8	9.4
Treatment for heart disease^b^	6.4	7.7

*BMI* body mass index, *DBP* diastolic blood pressure, *Na*/*K ratio* sodium/potassium ratio, *SBP* systolic blood pressure

^a^Means ± SD

^b^%

Table 3 shows the relationship between urinary Na/K ratio, BMI, alcohol consumption habit, use of diabetes medication, and treatment for heart disease and hypertension. Supplementary Table 2 shows the relationships of urinary Na/K ratio, BMI, alcohol consumption habit, use of diabetes medication and treatment for heart disease with hypertension in participants who did not take antihypertensive medication. In both 2017 and 2018, the urinary Na/K ratio was consistently and linearly associated with hypertension independent of age, sex, alcohol consumption habit, BMI, use of diabetes medication, and treatment for heart disease. The relationship was more significant in participants who did not take antihypertensive medication than in those who took antihypertensive medication.

**Table 3:** Relationship of Na/K ratio, drinking habit, BMI, use of diabetes medication, and treatment for heart disease with hypertension among 12,890 participants who underwent health checkup both in 2017 and in 2018. We analyzed the relationship between the urinary Na/K ratio and hypertension adjusted for age, sex, BMI, alcohol intake, use of diabetes medication and treatment for heart disease

2017 data (*n* = 12,877)		Odds ratio, 95% CI			2018 data (*n* = 12,877)		Odds ratio, 95% CI		
Age	per 1 year	1.08	1.07	1.08	Age	per 1 year	1.08	1.07	1.08
Women	vs men	1.11	1.01	1.21	Women	vs men	1.13	1.03	1.24
Na/K ratio	<3.0	Ref.	Ref.	Ref.	Na/K ratio	<3.0	Ref.	Ref.	Ref.
	3.0–3.9	1.03	0.90	1.18		3.0–3.9	0.94	0.83	1.07
	4.0–4.9	1.07	0.94	1.22		4.0–4.9	0.99	0.88	1.13
	5.0–5.9	1.14	0.995	1.32		5.0–5.9	1.15	1.01	1.32
	6.0–6.9	1.27	1.09	1.48		6.0–6.9	1.16	0.99	1.35
	7.0–7.9	1.53	1.28	1.82		7.0–7.9	1.39	1.16	1.67
	8.0–8.9	1.47	1.19	1.80		8.0–8.9	1.38	1.10	1.72
	9.0–9.9	2.02	1.54	2.64		9.0–9.9	1.71	1.29	2.28
	10.0–	2.16	1.79	2.60		10.0–	1.85	1.44	2.38
Drinking habit	Nondrinker	Ref.	Ref.	Ref.	Drinking habit	Nondrinker	Ref.	Ref.	Ref.
	<1 drink/day	1.14	1.04	1.26		<1 drink/day	1.10	1.00	1.21
	1.0–1.9 drink/day	2.07	1.81	2.37		1.0–1.9 drink/day	1.87	1.64	2.14
	≥2.0 drink/day	2.62	2.17	3.16		≥2.0 drink/day	3.06	2.52	3.72
BMI	per 1 kg/m^2^	1.18	1.16	1.19	BMI	per 1 kg/m^2^	1.18	1.17	1.20
User of diabetes medication	vs nonuser of diabetes medication	1.57	1.36	1.81	User of diabetes medication	vs nonuser of diabetes medication	1.51	1.31	1.74
Treatment for heart disease	vs without treatment for heart disease	1.21	1.03	1.44	Treatment for heart disease	vs without treatment for heart disease	1.55	1.32	1.81

*BMI* body mass index, 95% *CI* 95% confidence interval, *Na*/*K* ratio sodium/potassium ratio

In addition, because participants with new diabetes mellitus and heart disease may have changed their lifestyles, we further analyzed the relationship between urinary Na/K ratio and hypertension, excluding those with new onset diabetes mellitus or heart disease. Consequently, our results essentially remained unchanged (data not shown).

Table 4 shows the relationship between the change in SBP between 2017 and 2018 and the urinary Na/K ratio change, BMI change, and change in alcohol consumption volume adjusted for age and sex. Supplementary Table 3 shows the relationship between the change in SBP between 2017 and 2018 and the urinary Na/K ratio change, BMI change, and change in alcohol consumption volume adjusted for age and sex in participants who did not take antihypertensive medication. The change in SBP can be explained by older age, female sex, increase in alcohol consumption, increase in BMI, and an increase in urinary Na/K ratio. The finding was essentially unchanged between participants who did or did not use antihypertensive medication. In addition, the finding was unchanged when we further adjusted for SBP in 2017 (data not shown). The finding was consistent when we assessed DBP.

**Table 4:** Relationship between change in Na/K ratio and BP change using multiple regression analyses adjusted for age, sex, BMI change, and alcohol intake. We assessed the relationship between change in urinary Na/K ratio and BP change using multiple regression analyses adjusted for age, sex, BMI change, and alcohol intake

All participants (*n* = 12,877)					
SBP	*β*	*P* value	DBP	*β*	*P* value
Age	0.02	<0.01	Age	0.00	0.97
Sex	1.47	<0.01	Sex	0.98	<0.01
Difference between alcohol in 2017 and in 2018	1.41	<0.01	Difference between alcohol in 2017 and in 2018	0.73	<0.01
Difference between BMI in 2017 and in 2018	2.25	<0.01	Difference between BMI in 2017 and in 2018	1.26	<0.01
Difference between Na/K ratio in 2017 and in 2018	0.43	<0.01	Difference between Na/K ratio in 2017 and in 2018	0.22	<0.01

*BMI* body mass index, *BP* blood pressure, *DBP* diastolic blood pressure, *Na*/*K ratio* sodium/potassium ratio, *SBP* systolic blood pressure

Table 5 shows the difference in BP between the 2017 data and 2018 data according to the urinary Na/K ratio quartile in 2017. SBP decline was higher in participants in the high urinary Na/K ratio quartile. The finding was essentially unchanged when we excluded the use of antihypertensive medication.

**Table 5:** Mean and Standard deviation (SD) of change in lifestyle according to quartile of Na/K ratio in 2017. We analyzed the BP difference according to the urinary Na/K ratio quartile in 2017

Na/K ratio in 2017	<3.4 (*n* = 3,138)		3.5–4.7 (*n* = 3,060)		4.8–6.5 (*n* = 3,358)		≥6.6 (*n* = 3,321)	
	Mean	SD	Mean	SD	Mean	SD	Mean	SD
Difference in BMI in 2018 and 2017	0.0	0.8	0.0	0.8	0.0	0.8	0.0	0.8
Difference in Na/K ratio in 2018 and 2017	1.4	1.9	0.5	1.9	−0.4	2.1	−3.4	3.9
Difference in SBP in 2018 and 2017	−0.2	13.8	−0.7	14.0	−1.4	14.2	−2.4	14.4
Difference in DBP in 2018 and 2017	1.4	8.6	1.0	8.6	0.7	8.6	0.1	9.1
Difference in alcohol drinking in 2018 and 2017	0.0	0.4	0.0	0.3	0.0	0.3	0.0	0.4
SBP in 2017	129.0	17.6	131.2	17.3	132.8	18.0	135.2	18.0

*BMI* body mass index, *DBP* diastolic blood pressure, *Na*/*K ratio* sodium/potassium ratio, *SBP* systolic blood pressure, *SD* standard deviation

## Discussion

In this study, we observed a positive relationship between the urinary Na/K ratio and hypertension. Moreover, the change in urinary Na/K ratio was associated with the change in SBP and DBP. Compared to 2017, both SBP and the urinary Na/K ratio declined in 2018. Since this was a one-arm study, we could not confirm any causal relationship. However, the finding that the urinary Na/K ratio declined after informing the participants of their own urinary Na/K ratio suggested that measuring the urinary Na/K ratio and providing feedback to participants may potentially encourage behaviors such as salt intake reduction and increased vegetable intake. Consequently, this behavioral change might lower the average BP in a community.

According to the Japanese National Health and Nutrition Survey in 2010, 40% of respondents reduced their salt intake to prevent or improve lifestyle-related diseases, including hypertension [17], but there was no way of easily and immediately assessing their dietary Na balance. The introduction of the urinary Na/K ratio measurement assessed by a urinary Na/K ratio-measuring device in the health checkup site might provide the participants with an opportunity to review their dietary balance and therefore potentially change their lifestyle. Moreover, some participants who revisited the health checkup site in 2018 informed the public nurse that they changed their salt intake based on the 2017 results. It is expected that a change in personal lifestyle will result in BP reduction and subsequently lead to the prevention of other salt intake-related and BP-related diseases such as stroke, ischemic heart disease, and gastric cancer.

In this study, we observed a positive relation between urinary Na/K level and SBP independent of BMI and alcohol intake. This finding is consistent with previous studies [1–4]. Moreover, the change in urinary Na/K ratio was associated with a change in SBP and DBP [18]. Since a few prospective studies reported that the baseline urinary Na/K ratio was associated with future high BP, we reconfirmed these findings [19, 20]. In addition, we observed that the high baseline urinary Na/K ratio group was associated with a significant decline in BP compared with the findings in the low baseline urinary Na/K ratio group. Therefore, the effect of urinary Na/K ratio decline might be more effective in participants with a high urinary Na/K ratio. However, this relationship might be primarily due to strong regression to the mean effect on the urinary Na/K ratio. Repeated measurements of the urinary Na/K ratio showed a positive association with BP or hypertension [14, 21]. Further analysis with repeated casual urine samples might be required to clarify this issue.

In this study, we observed that the average SBP in our participants declined from 132.1 mmHg in 2017 to 130.9 mmHg in 2018, despite the fact that the participants aged 1 year. Multiple regression analyses showed that the SBP decline could be explained by BMI change, change in alcohol consumption habits, and change in urinary Na/K ratio. Since the proportion of participants who consumed more than one drink or had an average BMI was unchanged between 2017 and 2018, we considered that the SBP change in Tome City might be partially explained by a decline in the urinary Na/K ratio change. Another possible reason for BP decline was the change in outside temperature. The outside temperature increased slightly by 0.4 °C from 2017 to 2018, and our previous reports showed that a 1 °C increment in outside temperature corresponds to a 0.4 mmHg decline in home SBP [22].

The following are the strengths of our study. Although the participation rate was not perfect, almost all the participants who visited the health checkup site participated in this study. Furthermore, 80% of the participants who underwent health checkups participated in the 2018 health checkup. Thus, our finding was representative of participants undergoing health checkups in Tome City. Finally, we collected urine samples in the morning. The casual urinary Na/K ratio has a diurnal variation and is higher in the morning and evening than the 24-h urinary Na/K ratio [23]. In our study, we collected urine samples in the morning because Tome City asked participants to collect their urine sample after waking and undergo a health checkup early in the morning (6:30–9:30 a.m.). Therefore, we could consider the influence of diurnal variation in the urinary Na/K ratio.

There are some limitations to this study. As described above, there was no control group in the study. Although we observed a decline in the urinary Na/K ratio and SBP in 1 year, we could not conclude whether the measurement of urinary Na/K in the health checkup was directly responsible for this decline. Although we did not have evidence to support the positive effects on BP and overall health, such as a change in dietary patterns (stopping adding salt or soy sauce to food at table, reducing use of salt in cooking, increasing vegetable/fruit consumption, increasing physical activity to reduce BMI, etc.), we assumed that participants might have slightly changed their lifestyle, which may slightly change the SBP, and therefore, we concluded that measuring the urinary Na/K ratio in community settings had the potential to counteract hypertension in a population approach.

In conclusion, we observed that the change in urinary Na/K ratio was associated with hypertension and changes in SBP and DBP, and this can be explained by a change in alcohol consumption habits, BMI, and urinary Na/K ratio in this study. The SBP declined at a population level from 2017 to 2018, although the BMI level or proportion of participants who drank alcohol was unchanged. This can be explained partly by a change in urinary Na/K level. Therefore, measuring the urinary Na/K ratio in community settings has the potential to counteract hypertension in a population approach.

## Supplementary information

Supplementary Information
