# Supplementary material for: Sodium/potassium ratio change was associated with blood pressure change: possibility of population approach for sodium/potassium ratio reduction in health checkup
Source: Hypertens Res. 2020 Aug 17;44(2):225–31. doi: 10.1038/s41440-020-00536-7 (PMC7815510; doi:10.1038/s41440-020-00536-7)
Supplement: Supplementary file 1 — Supplementary Information [file 41440_2020_536_MOESM1_ESM.docx]

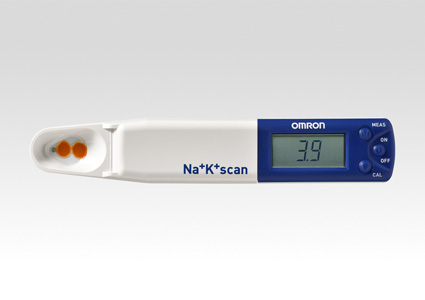


**Supplementary Figure 1**. Handy-sized urinary Na/K ratio monitors (HEU-001F; OMRON Healthcare Co., Ltd., Kyoto, Japan)


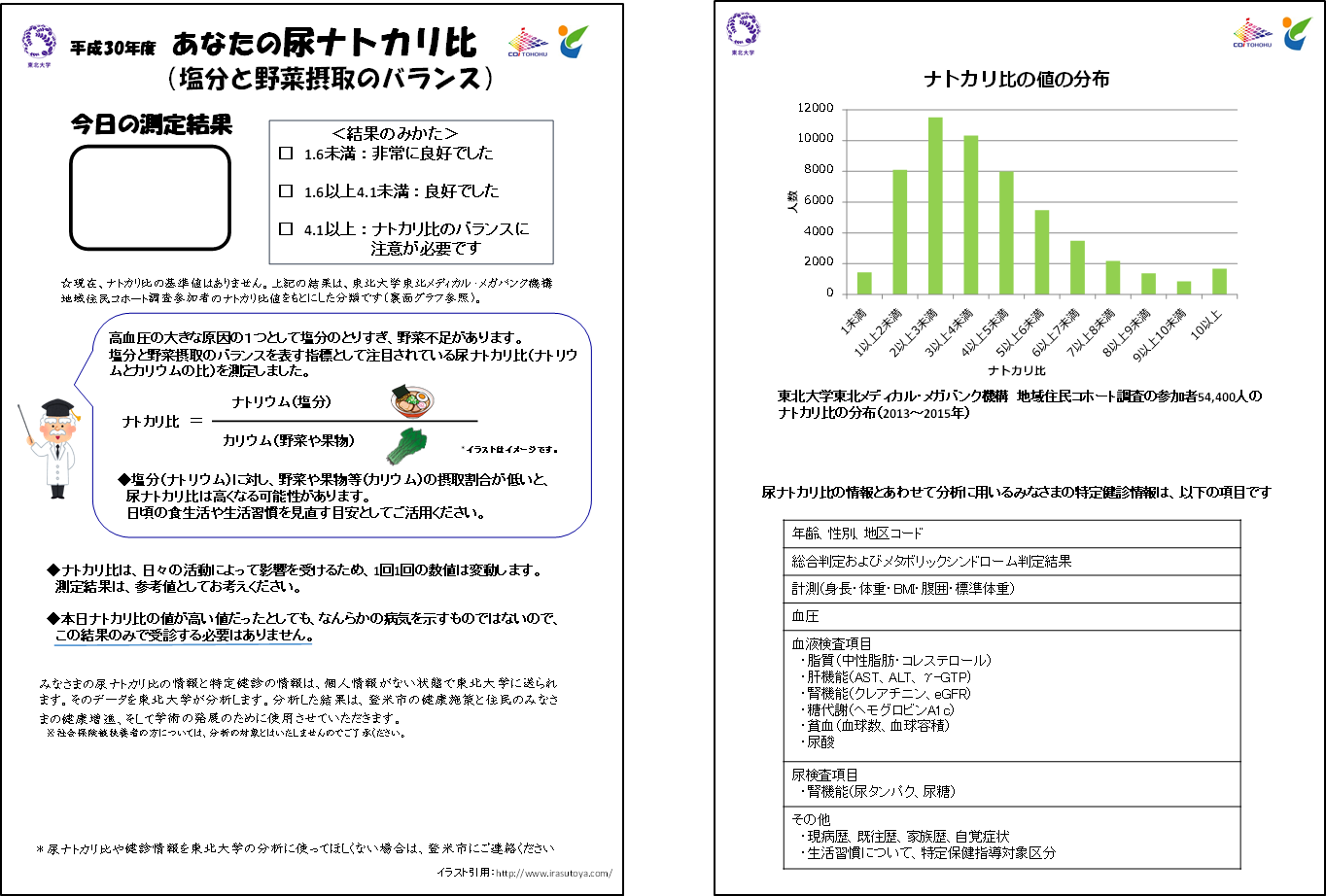


**Supplementary Figure 2**. List of feedback provided to the participants regarding the urinary Na/K ratio value

The urinary Na/K ratio value was recorded by the public nurse and the participants were provided feed-back with an explanatory material about the relationship between urinary Na/K ratio and BP.

**Supplementary Table1**. Characteristics of study participants who underwent health check-ups both in 2017 and 2018 according to urinary Na/K ratio, Tome Na/K Measuring Project, 2017-2018

| **Na/K ratio in 2017** | 0.0-0.9 | 1.0-1.9 | 2.0-2.9 | 3.0-3.9 | 4.0-4.9 | 5.0-5.9 | 6.0-6.9 | 7.0-7.9 | 8.0-8.9 | 9.0-9.9 | 10.0- |
| --- | --- | --- | --- | --- | --- | --- | --- | --- | --- | --- | --- |
| **n** | 46 | 534 | 1,490 | 2,214 | 2,401 | 1,950 | 1,476 | 961 | 598 | 335 | 872 |
| **Age (years)^a^** | 61.0 ± 15.3 | 61.8 ± 14.9 | 63.4 ± 14.4 | 64.6 ± 13.7 | 66.2 ± 12.7 | 66.8 ± 12.5 | 66.1 ± 12.9 | 66.5 ± 12.9 | 65.2 ± 12.9 | 65.6 ± 12.8 | 65.9 ± 13.0 |
| **Sex (women)^b^** | 41.3 | 48.7 | 54.2 | 52.7 | 53.8 | 52.9 | 51.8 | 49.0 | 45.8 | 39.4 | 39.7 |
| **BMI (kg/m^2^)^a^** | 23.7 ± 4.8 | 23.7 ± 3.5 | 23.7 ± 3.8 | 23.7 ± 3.6 | 23.7 ± 3.5 | 23.7 ± 3.6 | 23.7 ± 3.4 | 23.9 ± 3.7 | 24.1 ± 3.9 | 24.0 ± 3.8 | 24.0 ± 3.6 |
| **Non Drinker^b^** | 47.8 | 53.9 | 54.7 | 56.1 | 55.0 | 54.8 | 55.6 | 51.1 | 49.2 | 47.2 | 49.3 |
| **Alcohol < 1 drink/day^b^** | 21.7 | 24.0 | 27.9 | 26.6 | 26.5 | 26.2 | 28.2 | 28.8 | 27.8 | 25.1 | 28.4 |
| **Alcohol 1-2 drink/day^b^** | 21.7 | 15.5 | 12.1 | 12.2 | 12.7 | 14.8 | 11.7 | 14.1 | 13.7 | 19.1 | 16.1 |
| **Alcohol 2- drink/day^b^** | 8.7 | 6.6 | 5.3 | 5.1 | 5.7 | 4.2 | 4.5 | 6.0 | 9.4 | 8.7 | 6.2 |
| **Na/K ratio^a^** | 0.8 ± 0.2 | 1.6 ± 0.3 | 2.5 ± 0.3 | 3.5 ± 0.3 | 4.5 ± 0.3 | 5.4 ± 0.3 | 6.4 ± 0.3 | 7.4 ± 0.3 | 8.4 ± 0.3 | 9.4 ± 0.3 | 13.4 ± 3.2 |
| **SBP (mmHg)^a^** | 124.0 ± 15.6 | 128.8 ± 16.9 | 128.7 ± 18.0 | 130.1 ± 17.4 | 132.0 ± 17.4 | 132.5 ± 17.9 | 133.5 ± 18.0 | 134.6 ± 17.6 | 133.9 ± 17.8 | 136.3 ± 16.4 | 137.1 ± 19.2 |
| **DBP (mmHg)^a^** | 71.7 ± 10.1 | 73.3 ± 10.8 | 73.4 ± 10.9 | 73.8 ± 10.7 | 74.7 ± 10.7 | 75.0 ± 11.3 | 75.6 ± 10.8 | 76.4 ± 10.7 | 76.3 ± 11.5 | 78.2 ± 11.0 | 78.4 ± 12.2 |
| **User of antihypertensive Medication^b^** | 37.0 | 37.6 | 35.4 | 36.5 | 38.5 | 39.1 | 40.0 | 43.4 | 42.0 | 48.4 | 46.9 |
| **User of diabetes medication^b^** | 8.7 | 7.9 | 7.7 | 8.9 | 9.4 | 9.1 | 8.5 | 8.7 | 9.9 | 9.6 | 8.7 |
| **Treatment for heart disease^b^** | 8.7 | 6.7 | 5.0 | 5.8 | 6.0 | 7.1 | 7.2 | 6.2 | 5.7 | 7.8 | 8.4 |

| **Na/K ratio in 2018** | 0.0-0.9 | 1.0-1.9 | 2.0-2.9 | 3.0-3.9 | 4.0-4.9 | 5.0-5.9 | 6.0-6.9 | 7.0-7.9 | 8.0-8.9 | 9.0-9.9 | 10.0- |
| --- | --- | --- | --- | --- | --- | --- | --- | --- | --- | --- | --- |
| **n** | 47 | 559 | 1,585 | 2,510 | 2,685 | 2,102 | 1,385 | 834 | 482 | 294 | 394 |
| **Age (years)^a^** | 62.7 ± 15.3 | 63.5 ± 15.3 | 65.6 ± 15.3 | 66.3 ± 13.3 | 67.2 ± 12.8 | 66.7 ± 13.2 | 67.2 ± 13.2 | 66.9 ± 13.1 | 65.2 ± 13.5 | 67.1 ± 12.8 | 65.7 ± 13.0 |
| **Sex (women)^b^** | 38.3 | 52.4 | 54.8 | 53.7 | 53.3 | 50.4 | 48.7 | 47.1 | 43.6 | 40.1 | 38.6 |
| **BMI (kg/m^2^)^a^** | 23.0 ± 3.2 | 23.7 ± 3.8 | 23.9 ± 3.8 | 23.7 ± 3.7 | 23.6 ± 3.5 | 23.6 ± 3.7 | 24.0 ± 3.5 | 23.9 ± 3.7 | 24.1 ± 3.8 | 24.0 ± 3.4 | 24.2 ± 3.9 |
| **Non drinker^b^** | 53.2 | 56.7 | 56.9 | 55.6 | 55.0 | 53.8 | 53.1 | 52.0 | 50.4 | 46.6 | 45.7 |
| **Alcohol < 1 drink/day^b^** | 23.4 | 25.9 | 26.1 | 26.1 | 27.9 | 28.2 | 27.2 | 26.9 | 24.9 | 26.9 | 26.7 |
| **Alcohol 1-2 drink/day^b^** | 17.0 | 10.9 | 12.1 | 13.1 | 12.4 | 13.0 | 14.6 | 15.7 | 15.4 | 18.0 | 20.3 |
| **Alcohol 2- drink/day^b^** | 6.4 | 6.4 | 4.9 | 5.1 | 4.7 | 5.0 | 5.1 | 5.4 | 9.3 | 8.5 | 7.4 |
| **Na/K ratio^a^** | 0.8 ± 0.2 | 1.6 ± 0.3 | 2.5 ± 0.3 | 3.5 ± 0.3 | 4.4 ± 0.3 | 5.4 ± 0.3 | 6.4 ± 0.3 | 7.4 ± 0.3 | 8.4 ± 0.3 | 9.4 ± 0.3 | 11.7 ± 1.8 |
| **SBP (mmHg)^a^** | 125.7 ± 16.8 | 128.4 ± 16.7 | 128.3 ± 16.4 | 129.3 ± 17.2 | 130.0 ± 16.9 | 131.9 ± 17.9 | 132.9 ± 17.8 | 133.7 ± 18.4 | 134.2 ± 17.8 | 135.7 ± 17.3 | 135.5 ± 17.9 |
| **DBP (mmHg)^a^** | 74.1 ± 12.9 | 75.1 ± 11.1 | 74.7 ± 10.4 | 74.8 ± 10.9 | 75.2 ± 10.4 | 76.1 ± 10.8 | 77.1 ± 11.3 | 77.0 ± 11.8 | 78.1 ± 11.0 | 78.5 ± 10.9 | 79.1 ± 11.1 |
| **User of antihypertensive Medication^b^** | 31.9 | 40.4 | 40.5 | 39.7 | 41.2 | 40.9 | 42.8 | 44.8 | 44.2 | 48.6 | 50.0 |
| **User of diabetes medication^b^** | 6.4 | 9.7 | 9.7 | 9.0 | 9.4 | 9.4 | 9.0 | 10.8 | 11.4 | 8.5 | 6.6 |
| **Treatment for heart disease^b^** | 17.0 | 8.2 | 6.4 | 7.8 | 8.3 | 6.6 | 8.5 | 7.1 | 7.7 | 6.5 | 10.7 |

BMI, body mass index; DBP, diastolic blood pressure; Na/K ratio, sodium/potassium ratio; SBP, systolic blood pressure

^a^ means ± SD

^b^ %

**Supplementary Table2**. Relationship of Na/K ratio, drinking habit, BMI, use of diabetes medication, and treatment for heart disease with hypertension in participants who did not take antihypertensive medication

| **2017 data without user of antihypertensive medication (n=7,808)** | | **Odds ratio, 95%CI** | | |  | **2018 data without user of antihypertensive medication (n=7,512)** | | **Odds ratio, 95%CI** | | |
| --- | --- | --- | --- | --- | --- | --- | --- | --- | --- | --- |
| **Age** | **per 1 year** | 1.05 | 1.04 | 1.05 |  | **Age** | **per 1 year** | 1.05 | 1.04 | 1.05 |
| **Women** | **vs men** | 0.98 | 0.86 | 1.11 |  | **Women** | **vs men** | 1.07 | 0.94 | 1.22 |
| **Na/K ratio** | **< 3.0** | Ref. | Ref. | Ref. |  | **Na/K ratio** | **< 3.0** | Ref. | Ref. | Ref. |
|  | **3.0-3.9** | 1.22 | 0.997 | 1.50 |  |  | **3.0-3.9** | 0.97 | 0.80 | 1.19 |
|  | **4.0-4.9** | 1.30 | 1.06 | 1.58 |  |  | **4.0-4.9** | 1.09 | 0.89 | 1.32 |
|  | **5.0-5.9** | 1.50 | 1.22 | 1.84 |  |  | **5.0-5.9** | 1.41 | 1.15 | 1.73 |
|  | **6.0-6.9** | 1.64 | 1.32 | 2.05 |  |  | **6.0-6.9** | 1.44 | 1.15 | 1.80 |
|  | **7.0-7.9** | 1.99 | 1.56 | 2.54 |  |  | **7.0-7.9** | 1.74 | 1.34 | 2.26 |
|  | **8.0-8.9** | 1.96 | 1.48 | 2.61 |  |  | **8.0-8.9** | 1.69 | 1.23 | 2.33 |
|  | **9.0-9.9** | 2.52 | 1.76 | 3.63 |  |  | **9.0-9.9** | 2.10 | 1.43 | 3.09 |
|  | **10.0-** | 2.87 | 2.24 | 3.69 |  |  | **10.0-** | 2.23 | 1.57 | 3.17 |
| **Drinking habit** | **Nondrinker** | Ref. | Ref. | Ref. |  | **Drinking habit** | **Nondrinker** | Ref. | Ref. | Ref. |
|  | **< 1 drink/day** | 1.12 | 0.98 | 1.28 |  |  | **< 1 drink/day** | 1.08 | 0.94 | 1.24 |
|  | **1.0-1.9 drink/day** | 1.78 | 1.49 | 2.13 |  |  | **1.0-1.9 drink/day** | 1.53 | 1.26 | 1.85 |
|  | **≥　2.0 drink/day** | 2.26 | 1.76 | 2.89 |  |  | **≥　2.0 drink/day** | 2.85 | 2.20 | 3.68 |
| **BMI** | **per 1 kg/m^2^** | 1.14 | 1.12 | 1.15 |  | **BMI** | **per 1 kg/m^2^** | 1.14 | 1.12 | 1.16 |
| **User of diabetes medication** | **vs non-user of diabetes medication** | 0.79 | 0.63 | 1.00 |  | **User of diabetes medication** | **vs non-user of diabetes medication** | 0.57 | 0.44 | 0.75 |
| **Treatment for heart disease** | **vs without treatment for heart disease** | 0.44 | 0.32 | 0.60 |  | **Treatment for heart disease** | **vs without treatment for heart disease** | 0.47 | 0.34 | 0.65 |

BMI, body mass index; 95% CI, 95% confidence interval; Na/K ratio, sodium/potassium ratio

**Supplementary Table3**. Relationship between change in Na/K ratio and BP change using multiple regression analyses adjusted for age, sex, BMI change, and alcohol intake in participants who did not take antihypertensive medication.

| **Non-user of antihypertensive medication both in 2017 and in 2018 (n=7,356)** | | | | | | | |  | | |  | | |
| --- | --- | --- | --- | --- | --- | --- | --- | --- | --- | --- | --- | --- | --- |
| **SBP** | **β** | **P value** |  | | **DBP** | | **β** | | | **P value** | | |  |
| Age | 0.04 | < 0.01 |  | Age | | 0.00 | | | 0.63 | | |  |  |
| Sex | 0.95 | < 0.01 |  | Sex | | 0.77 | | | < 0.01 | | |  |  |
| Difference between alcohol in 2017 and in 2018 | 0.73 | 0.10 |  | Difference between alcohol in 2017 and in 2018 | | 0.13 | | | 0.66 | | |  |  |
| Difference between BMI in 2017 and in 2018 | 2.34 | < 0.01 |  | Difference between BMI in 2017 and in 2018 | | 1.31 | | | < 0.01 | | |  |  |
| Difference between Na/K ratio in 2017 and in 2018 | 0.49 | < 0.01 |  | Difference between Na/K ratio in 2017 and in 2018 | | 0.25 | | | < 0.01 | | |  |  |

BMI, body mass index; BP, blood pressure, DBP, diastolic blood pressure; Na/K ratio, sodium/potassium ratio; SBP, systolic blood pressure
